# In silico design of peptide inhibitors for Dengue virus to treat Dengue virus-associated infections

**DOI:** 10.1038/s41598-024-63064-1

**Published:** 2024-06-07

**Authors:** Amar Ajmal, Muhammad Shahab, Muhammad Waqas, Guojun Zheng, Maryam Zulfat, Yousef A. Bin Jardan, Gezahign Fentahun Wondmie, Mohammed Bourhia, Ijaz Ali

**Affiliations:** 1 Department of Biochemistry, Abdul Wali Khan University Mardan, Mardan, Pakistan; 2 State Key Laboratories of Chemical Resources Engineering, Beijing University of Chemical Technology, Beijing, 100029 People’s Republic of China; 3 Natural and Medical Sciences Research Center, University of Nizwa, Birkat Al-Mouz, 616 Nizwa, Oman; 4 Department of Pharmaceutics, College of Pharmacy, King Saud University, P.O. Box 11451, Riyadh, Saudi Arabia; 5 Department of Biology, Bahir Dar University, P.O.Box 79, Bahir Dar, Ethiopia; 6 Department of Chemistry and Biochemistry, Faculty of Medicine and Pharmacy, Ibn Zohr University, 70000 Laayoune, Morocco; 7 Centre for Applied Mathematics and Bioinformatics, Gulf University for Science and Technology, Hawally, Kuwait

**Keywords:** Peptide inhibitors, Residue scanning, MD simulation, Binding energy calculation, Biotechnology, Drug discovery

## Abstract

Dengue virus is a single positive-strand RNA virus that is composed of three structural proteins including capsid, envelope, and precursor membrane while seven non-structural proteins (NS1, NS2A, NS2B, NS3A, NS3B, NS4, and NS5). Dengue is a viral infection caused by the dengue virus (DENV). DENV infections are asymptomatic or produce only mild illness. However, DENV can occasionally cause more severe cases and even death. There is no specific treatment for dengue virus infections. Therapeutic peptides have several important advantages over proteins or antibodies: they are small in size, easy to synthesize, and have the ability to penetrate the cell membranes. They also have high activity, specificity, affinity, and less toxicity. Based on the known peptide inhibitor, the current study designs peptide inhibitors for dengue virus envelope protein using an alanine and residue scanning technique. By replacing I21 with Q21, L14 with H14, and V28 with K28, the binding affinity of the peptide inhibitors was increased. The newly designed peptide inhibitors with single residue mutation improved the binding affinity of the peptide inhibitors. The inhibitory capability of the new promising peptide inhibitors was further confirmed by the utilization of MD simulation and free binding energy calculations. The molecular dynamics simulation demonstrated that the newly engineered peptide inhibitors exhibited greater stability compared to the wild-type peptide inhibitors. According to the binding free energies MM(GB)SA of these developed peptides, the first peptide inhibitor was the most effective against the dengue virus envelope protein. All peptide derivatives had higher binding affinities for the envelope protein and have the potential to treat dengue virus-associated infections. In this study, new peptide inhibitors were developed for the dengue virus envelope protein based on the already reported peptide inhibitor.

## Introduction

The finding of insulin, a peptide consisting of 51 amino acids, is a highly notable breakthrough in the field of drug discovery research^1,2^. The FDA has approved approximately 80 therapeutic peptides for human use, and there are now hundreds more undergoing clinical testing. Peptide drugs have proven to be effective in the treatment of cancer, infectious illnesses, and cardiovascular disease^3^. The main advantages of therapeutic peptides over antibodies include their small size, simplicity in synthesis, and capacity to cross cell membranes. The Dengue virus causes dengue infections, each year an estimated 284 to 528 million infections worldwide are caused by the dengue virus^4^. Nearly 98 million of these cases are clinically apparent. According to a report 2.5 billion individuals reside in high-risk areas, making dengue infection a significant public health issue^5^. Drugs and vaccines are not available for this infection at the moment. The immune system responds protectively to the infectious serotype during the initial exposure, but recurrent exposure to heterologous serotypes can result in serious illness. Asymptomatic to severe hemorrhage or death is the clinical disease spectrum^6–10^. Four different serotypes of the enveloped flavivirus known as dengue virus (DENV) affect people. It is a member of the Flaviviridae genus. Dengue virus is a single positive-strand RNA virus that is composed of three structural proteins including capsid, envelope, and precursor membrane while seven non-structural proteins (NS1, NS2A, NS2B, NS3A, NS3B, NS4, and NS5^11,12^. Numerous human cell types, including kidney cells, monocytes, hepatocytes, macrophages, and splenocytes, are susceptible to infection by this virus. The fusing of the viral and cellular membranes is one of the fundamental events for enveloped viruses like DENV^13–15^. Three domains and a final stem region make up the DENV envelope protein^16^. The entry of viruses into human host cells involves receptors like CD44, CD206, and CD209^5,15^. Following initial recognition, the virus enters the cell through endocytosis, when the E protein undergoes a conformational shift. Domain I is one of the central domains that serves as a hinge, Domain II helps in the initial attachment with the cell membrane and domain III adjusts its spatial distribution and exposes the stem area, allowing the cell membrane to connect to the viral membrane and resulting in the creation of a fusion pore. This enables the viral capsid and the positive polarity RNA to enter. High replication efficiency is made possible by the ability of this genetic material to serve as messenger RNA, which enters cell ribosomes directly^12,14,17,18^.

Due to their ability to mimic the surface of proteins and compete with them for binding, peptides can efficiently target protein–protein interactions. Furthermore, peptides can be readily produced and their characteristics can be altered using chemical procedures^19^. Peptide inhibitors are less harmful and are simple to synthesize and modify^20^. The DN59 peptide (MAILGDTAWDFGSLGGVFTSIGKALHQVFGAIY) is a 33 amino acid peptide initially reported by Hrobowskiet al and it was found that DN59 induces 99% inhibition of the Dengue virus Envelope protein at a 10 µM concentration^21,22^. Different Insilico methodologies are widely used for developing peptide inhibitors. Previously Shahab et al. used a residue scan approach for the development of peptide inhibitors for the shp2, and CIB1 complexes^23,24^. Similarly, Abbas et al. utilized Insilico tools for the designing of peptide inhibitors for 3CLpro of SARS-CoV-2^25^. In current study, a previously reported 33 amino acids long peptide inhibitor DN59 (MAILGDTAWDFGSLGGVFTSIGKALHQVFGAIY) was repurposed, and the new and promising peptide inhibitors were developed for the DNV based on the reported peptide inhibitor. By using MD simulation and free binding energy calculations, the inhibitory potential of the new promising peptide inhibitors was further validated. This research lays the foundations for the development of new peptide inhibitors that target the DNV envelope protein and can be helpful to combat dengue virus-associated infections.

## Method

### Peptide modeling

The amino acid sequence of the peptide was provided as input for the PEP-FOLD3 server. A total of five models were provided by the PEP-FOLD3 server^26^. The predicted peptide structures were used for validation by the ERRAT and Ramachandran plot analysis^27^. Only the validated model was used for further study.

### Protein-peptide docking

The 3D structure of the Envelope protein PDB ID 7A3Q was retrieved from the PDB database. The attached water molecules and the co-crystal ligands were eliminated from the structure. The energy minimization of the Envelope protein was carried out using an RMS gradient of 0.05^28^. Docking was performed using MOE (2016) to predict the interactions between the modeled peptide and the Envelope protein of the DNV^26^. The protein-peptide complex that revealed good binding score and interactions was used for further study.

### Alanine scanning

For designing a peptide inhibitor, it is crucial to have a thorough understanding of the exact binding interface of two proteins that are interacting with each other. The crucial residues implicated in the interactions at the Envelope-DN59 complex interface were identified. The dAffinity and dStability scores were calculated using the alanine scanning module (ASM) of the MOE software. The differences in binding energy that arise when an amino acid undergoes a mutation to alanine are denoted by the values of dAffinity and dStability^23^. In order to understand the impact of each amino acid mutated contributed to the stability of peptide the alanine scanning was carried out.

### Residue scanning

The residue scanning was conducted utilizing the residue scanning function within the MOE (2016) software^29^. The dAffinity and dStability scores that determine the relative change in the binding energy of the mutated peptides were calculated during residue scanning. A database of mutant peptides along with the stability and dstability and affinity and dAffinity was predicted after the completion of the residue scanning.

### New peptide library generation

The peptide inhibitors were obtained using a decoy strategy by altering the non-interacting residues of the peptide inhibitors were modified to other amino acids in order to enhance the affinity of the peptide-protein complex. A novel repertoire of peptides was produced by inducing mutations in the non-essential residues of the original peptide inhibitor^30^. The native-to-native mutations were eliminated to reduce the computation costs^29,31^. Finally, MD simulation was performed on the top five Envelope protein/decoy peptide complexes with the highest dAffinity and dStability scores. The native peptide/Envelope protein complex was simulated as a control system.

### MD simulation

An extensive MD simulation was performed in order to determine the stability of the native peptide and the top four generated peptides that had good dAffinity and dStability scores bound to the DNV Envelope protein. MD simulation studies for 100 ns were performed using AMBER v22^32^. The ff14SB force field was used in this study. The TIP3P water model was used to solve each system. The systems were neutralized by introducing the chloride (Cl^−^) ions^33^. The Particle Mesh Ewald approach was used to handle long-range electrostatic interactions. The energy of each system was minimized using 8000 cycles of the steepest descent and 4000 cycles of the conjugate gradient minimization algorithm^34^. The Langevin dynamics method was used to regulate the temperature. The hydrogen bonds were constrained using the SHAKE algorithm. Each minimized system underwent an equilibration of 200 ps. Then at a constant temperature and pressure, 100 ns MD simulation was carried out using the PMEMD. CUDA^35^. All the trajectories were examined using the CPPTRAJ module of AMBER software. For graphical representation, the Origin and PyMol software was used^36^.

### Principal component analysis (PCA)

To gain insights about the primary movement during the MD trajectory, PCA analysis was carried out. The covariance matrix was initially measured using the Cpptraj package by extracting the eigenvalues/vectors. The eigenvalue denotes mean square fluctuation, whereas PCs were used to depict the direction of large amplitude primary movement^37^. PC1 and PC2 were plotted for every complex. Principal Component Analysis (PCA) was performed to extract essential motions, with the top K eigenvectors capturing a significant portion of the total variance. The projection of the original data onto the selected principal components (PCi) was expressed as:

$$PCi \, = \sum_{j} = 1 \, vij \cdot xj$$

where M is the number of selected eigenvectors, v_ij_ is the j^th^ component of the i^th^ eigenvector, and x_j_ is the j^th^ coordinate of the original data. These analyses, in line with the methodology outlined by shahab et al.^24^, enabled a comprehensive exploration of the system structural dynamics, shedding light on its functional intricacies.

### Binding free energy calculation

The binding free energy (BFE) between the wild-type and mutant systems was calculated using the MMPBSA.Py script^38^. For the purpose of calculating BEF, the last 500 snapshot samples were used. For the binding free energy calculation, the following equation was used.

$$\Delta Gbind = \Delta Gcomplex - [\Delta Greceptor + \Delta Gligand]$$

Here, the term DGbind denotes the total binding energy, and the other terms in the equation denote the free energies of the complex, receptor, and ligand.

## Result and discussion

### Peptide inhibitor modeling and docking

**Figure 1:**
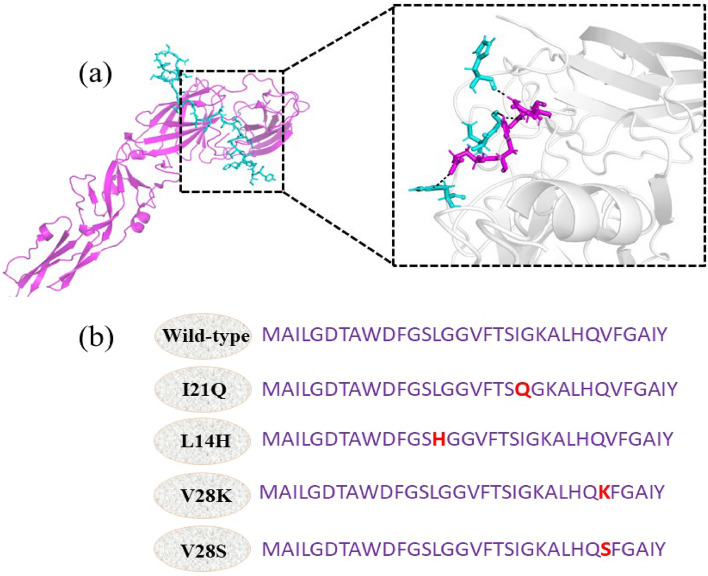
The binding interface of the peptide inhibitor docked with the enveloped protein.

The sequence of peptide inhibitors taken from the literature was uploaded to PepFold3 software and modeled. The PepFold3 server provided a total of five models of the peptide inhibitor. The model was then validated by PROCHECK server. Figure S1 shows the Ramachandran plot for the developed model and Fig. S2 shows the ERRAT plot for the model. According to Ramachandran plot analysis 76% of the residues were found in the most favored region, 24% of the residues were found in the additional allowed region while 0% of the residues were found in the disallowed region. ERRAT plot indicates an overall quality of the model as 100. The 3D structure of the peptide inhibitor was then docked with the envelope protein of the dengue virus. A total of 10 poses were generated. All the poses were ranked based on the docking score. The docking score of the complex was predicted as − 11.90 kcal/mol. The pose that revealed good docking score and interaction was then used for further study. Figure 1 shows the peptide inhibitor docked with the enveloped protein. The wild-type peptide inhibitors formed a total of seven hydrogen bonds with the envelope protein.

### Candidate peptide inhibitor generation

In this work, the dAffinity (changes in binding affinities) of the original peptide and the new peptide derivatives were calculated using the residue scanning function of the MOE software. The reference peptide's non-essential residues that have no role in interacting with the E protein were found by the alanine scanning approach and substituted with different residues using a process called residue scan mutagenesis. When mutated to alanine, several residues (I21, L14, G16, V17, T19, Q27, V28, and F29) have positive dAffinity values. In order to improve the proposed peptides' binding affinity for the E protein, these residues were substituted with other residues. To create a peptide library, only residues that did not interact with E protein were selected for substitution. Some single-residue substitutions were found to have significantly negative dAffinity values when compared to the reference peptide. A native-to-specific mutation that has raised the binding affinity for the receptor is indicated by a negative dAffinity score. By mutating a few residues, the suggested peptides' affinity for the envelope protein was improved (Table 1). By replacing I21 with Q21, L14 with H14, and V28 with K28 the binding affinity of the peptide inhibitors was increased. The newly designed peptide inhibitors with single residue mutation that improved binding affinity are listed in Table 1. To determine how these alterations affected complex stability, MD simulation was performed on the top four peptide complexes.

**Table 1:** Designed peptide inhibitors with reference to wild-type peptide.

S. No.	Peptide	Affinity kcal/mol)	dAffinity (kcal/ mol)
1	I21Q	− 95.2	− 2.56
2	L14H	− 98.0	− 2.25
3	V28K	− 98.7	− 1.86
4	V28S	− 98.5	− 1.67
5	Wild-type	− 89.6	− 0.89

### Stability and estimated half-life of the peptide inhibitors

The ProtParam server (http://web.expasy.org/protparam/) was used to calculate the molecular weight (MW) and stability of the designed peptide inhibitors^39^. Table 2 shows the molecular weight, estimated half-life, and instability index of the newly designed peptide inhibitors. A protein whose instability index is smaller than 40 is predicted as stable^40^. All the peptide inhibitors showed an instability index of less than 40 indicating that all the peptide inhibitors were stable.

**Table 2:** Instability index analysis, molecular weight and estimated half-life analysis.

S. No.	Peptide sequence	Molecular weight	Half-life ( h)	Instability index
I21Q	MAILGDTAWDFGSLGGVFTSQGKALHQVFGAIY	3458.94	30	14.58
L14H	MAILGDTAWDFGSHGGVFTSIGKALHQVFGAIY	3467.95	30	5.60
V28K	MAILGDTAWDFGSLGGVFTSIGKALHQKFGAIY	3473.01	30	11.03
V28S	MAILGDTAWDFGSLGGVFTSIGKALHQSFGAIY	3431.91	30	24.34
Wild	MAILGDTAWDFGSLGGVFTSIGKALHQVFGAIY	443.96	30	8.74

### RMSD analysis

**Figure 2:**
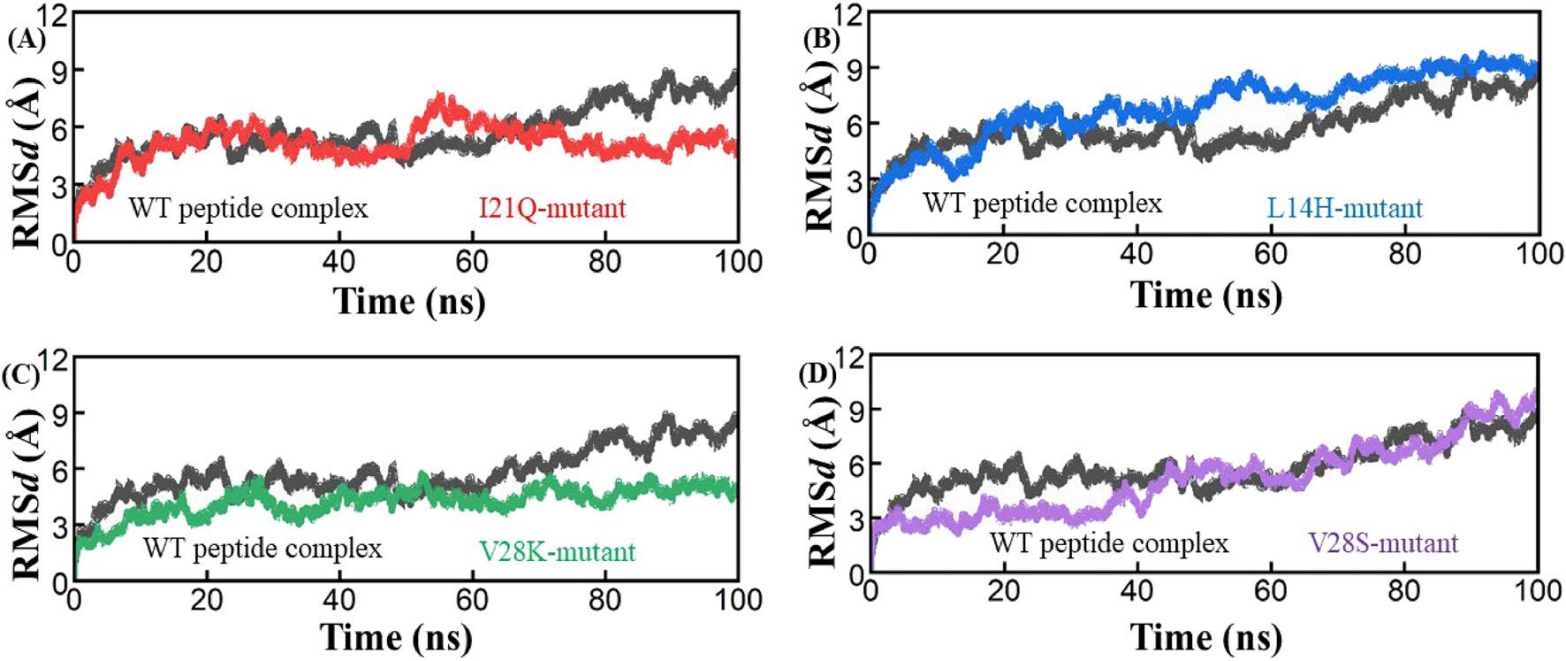
RMSd plots for the wild type (Black), I21Q (Red), L14H (Blue), V28K (green), and V28S (purple) peptide-protein complexes. The RMSd value is present on the Y-axis.

Root Mean Square Deviation (RMSD) are usually used to determine the stability of the receptor-ligand complex in MD simulations. In the present study, we performed explicit MD simulations of the most potent peptide inhibitors with their respective target, which is the envelope protein. The structural drift and stability were measured by plotting the root-mean-square deviation (RMSD) of protein Cα atoms as a function of time. All systems remained significantly stable over the course of the production run (Fig. 2). The RMSD plot in the WT peptide complex was stable from the beginning with a slight increase and decrease in the RMSD value but after 61 ns the RMSD reached its maximum value but then remained stable until the end of the simulation. In the I21Q complex, the RMSD plot was observed to be stable from the beginning and then remained unchanged for 50 ns. Between 50 and 60 ns, the RMSD increases slightly but then immediately decreases to its initial value for the entire simulation runtime (Fig. 2A). The L14H mutant complex RMSD plot shows a slightly higher RMSD value than the wild-type peptide complex but remained stable until the end of the MD run (Fig. 2B). The V28K mutant complex shows a static pattern of deviation as shown in Fig. 2C and remains stable throughout the simulation time. The V28S mutant complex showed that the RMSD plot was stable from 0 to 38 ns and then increased very slowly and slightly and the RMSd value reached from 3 to 9 Å (Fig. 2D). The observation is further supported by RMSF analysis which highlights the decreased mobility of active site residues in the case of peptide–bound complexes.

### Residual flexibility analysis

**Figure 3:**
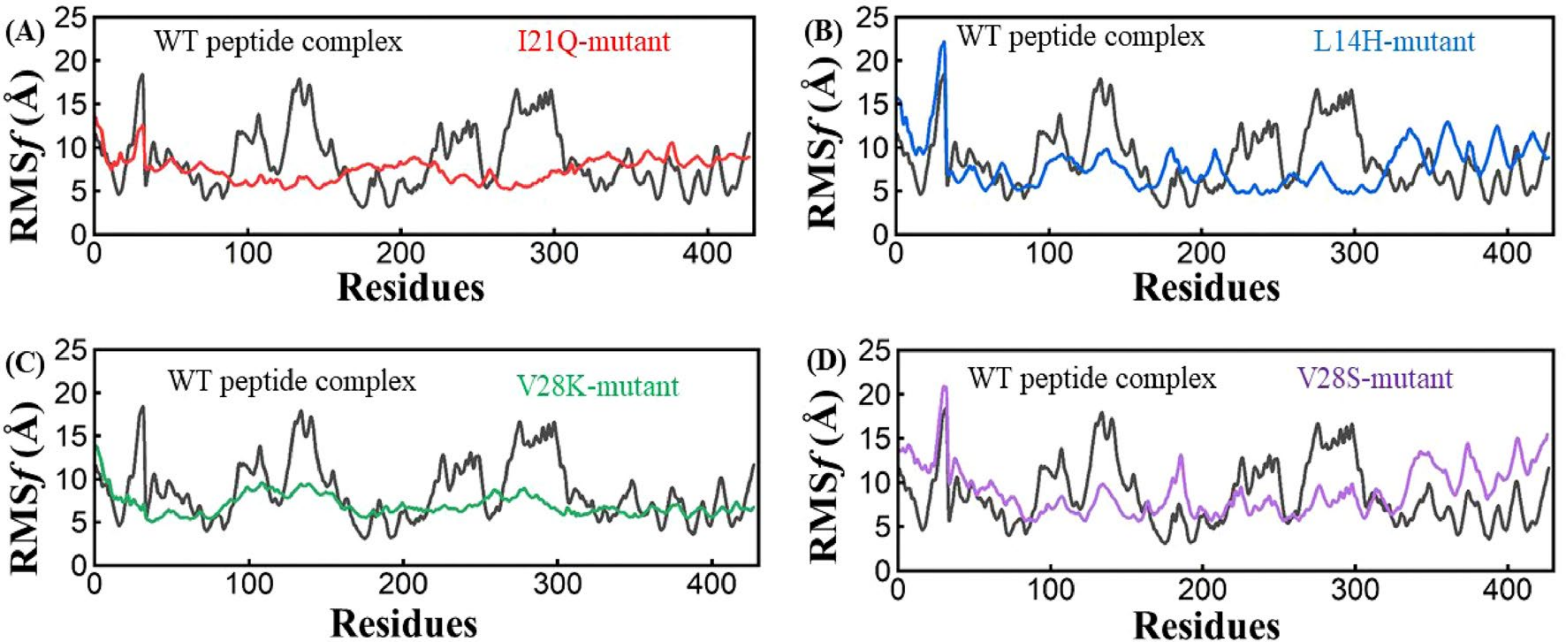
Root mean square deviation (RMSF) plots for the wild type (Black), I21Q (Red), L14H (Blue), V28K (green), and V28S (purple) peptide-protein complexes.

To examine peptide binding-mediated impacts on the structural flexibility of Envelope protein, root-mean fluctuations (RMSF) were computed by using the coordinates of the Cα atoms. The RMSF graph showed that the residues of the reference complex exhibit the highest instability at positions 1–25 (**∼**9.0 Å), 90–110 (**∼**15.0 Å), 120–150(**∼**18.5 Å), 210–260 (**∼**14.0 Å) and 270–310 (**∼**17.0 Å) in comparison to the mutated peptide complexes. However, I21Q mutant shows less fluctuations in MD simulation. The L14H mutant complex shows the highest fluctuation at positions 17–25 (∼23.0 Å) followed by a very slight increase and decrease in the RMSF plot. In the third mutant complex V28K, the RMSF graph showed no major fluctuations indicating structural stability. The V28S mutant complex gave the same result as L14H, initially showing a high peak at positions 15–19 followed by a slight fluctuation in the plot. In addition, the root mean square fluctuation (RMSF) analysis was performed based on residues indicated that all the designed peptides exhibited lower fluctuation in the binding sites compared to the reference peptide (Fig. 3).

### Hydrogen bonding interaction analysis from simulation trajectory

**Figure 4:**
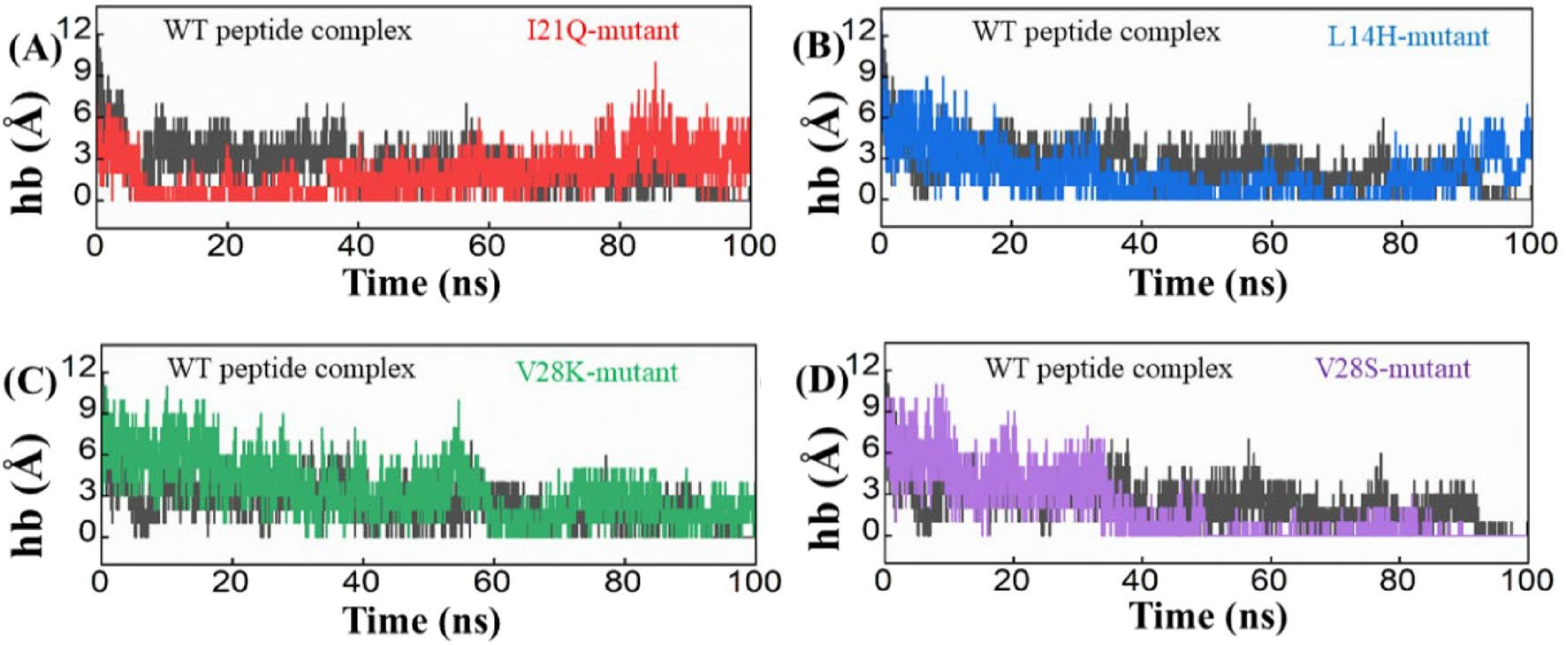
Hydrogen bond (Hb) analysis plots for the wild type (Black), I21Q (Red), L14H (Blue), V28K (green), and V28S (purple) peptide-protein complexes.

Stable hydrogen bonds are crucial for molecular recognition. In order to assess the stability of hydrogen bonds, it is necessary to employ molecular dynamics simulations, as comparing crystal structures alone is insufficient. Hydrogen bonding contact profile were analyzed from the simulation trajectory of all four designed peptide complexes and wild type complex of envelope protein (Fig. 4). According to hydrogen bond analysis, the H-bond connections between the peptide inhibitors we designed and the envelope protein are stronger than those between the wild type and the inhibitor. According to our research, generated peptides show a strong affinity for the envelope protein and may find application in therapy.

### Radius of Gyration

**Figure 5:**
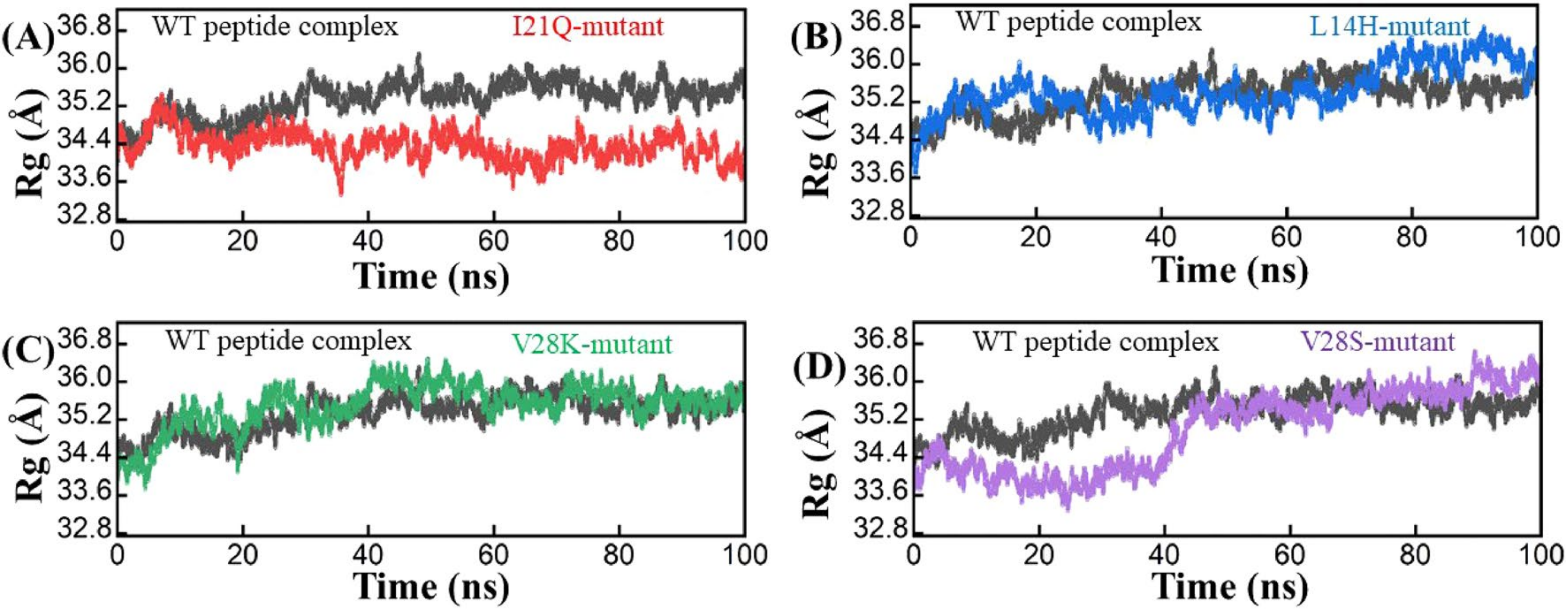
Radius of gyration (Rg) plots for the wild type (Black), I21Q (Red), L14H (Blue), V28K (green), and V28S (purple) peptide-protein complexes. The Rg value is present on the Y-axis.

To understand the structural compactness, the radius of gyration (Rg) was calculated. The wild-type Rg value was initially 35.2 Å, but at 30 ns the Rg value gradually changed to 36.0 Å and then stabilized at the end of the simulation. The I21Q mutant shows an Rg value similar to that of the wild type from the beginning up to 10 ns, but after 10 ns the Rg value drops from 35.2 Å to 34.4 Å and shows a lower average Rg value than the wild-type complex. The L14H mutant complex shows a lower Rg graph than the wild type, but after 75 ns the value of Rg increases from that of the wild-type complex. A third mutant complex, V28K, experienced a slight increase and decrease in Rg between 5 and 50 ns and then exhibited the same pattern of Rg as the wild-type complex. The V28S mutant complex shows a small Rg value of about 33.6 Å from the beginning to 40 ns, but a large deviation was observed after 40 ns and the plot reached 35.2 Å and remained constant until the end of the simulation. The Rg of each designed and wild type complex was displayed in Fig. 5.

### Analysis of conformational dynamics of top four Peptides

**Figure 6:**
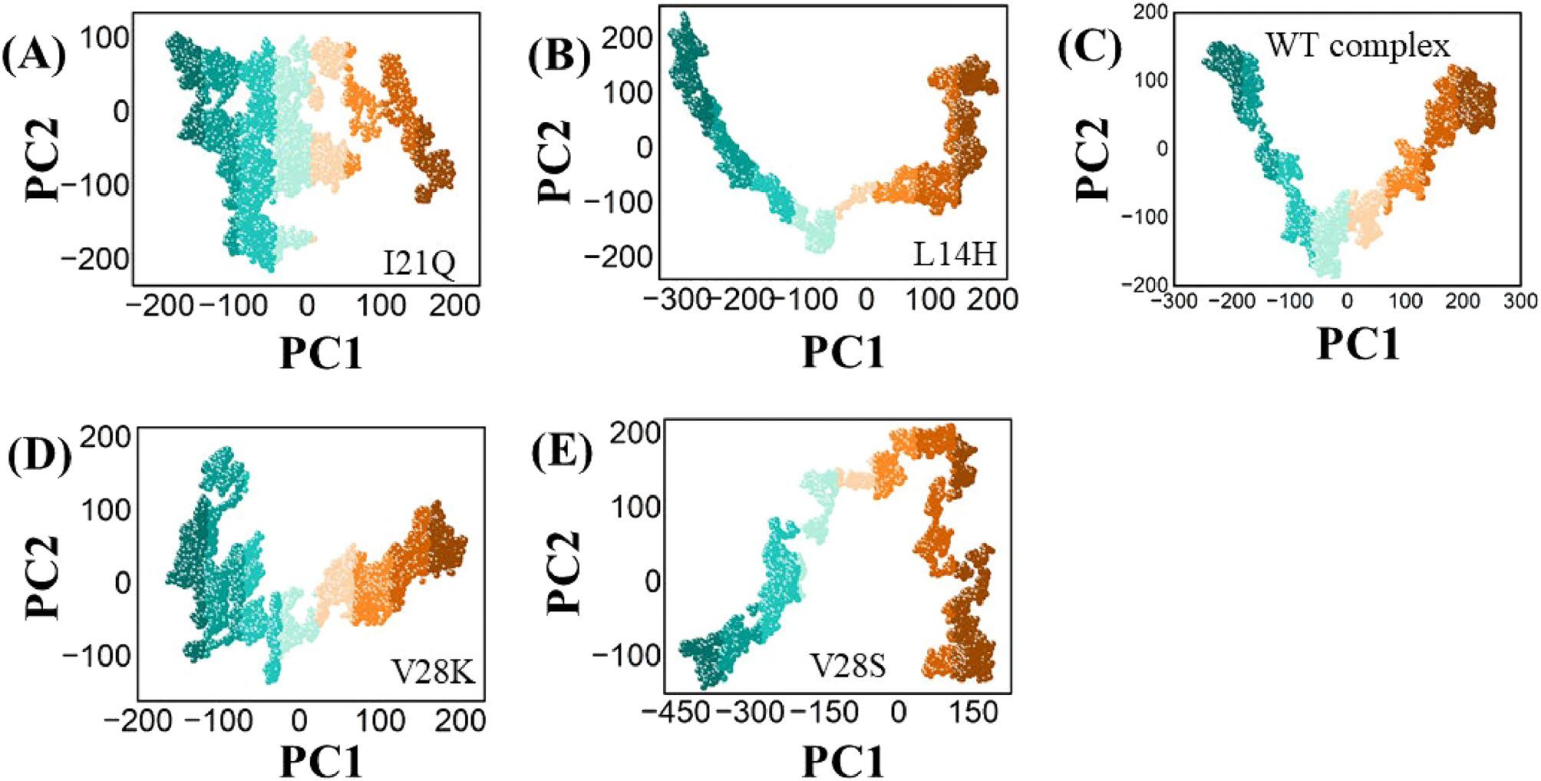
Principal component analysis (PCA) plots for the I21Q (**A**), L14H (**B**), and WT complex (**C**), and V28K (**D**) and V28S (**E**), peptide-protein complexes.

To understand the conformational dynamics of the reference peptide complex and the designed peptide complex, principal component analysis was performed using the AMBER v22 program. The fluctuation of the envelope protein is represented by its principal modes, which also indicate its direction (eigenvector) and magnitude (eigenvalue). PCA (Principal Component Analysis) contributes to characterizing the dynamic behavior of protein movement trajectories during MD simulations. Projection of top two PCA modes (PC1 and PC2) from reference peptide and designed peptides were plotted against 100 ns MD simulated conformation (Fig. 6). In order to define a unique conformation of the simulated system, PC1 and PC2 projections on the X and Y-axes, respectively, are essential subspaces that overlap with structurally similar conformations. It was observed that the protein clusters in the I21Q system, which were separated by the substrate from light blue to dark brown, had greater distribution and more local motions. The PC1 and PC2 regions are covered by dots in the I21Q system, extending from blue dots to dark brown dots with − 200 and + 200 and − 200 and + 100, respectively. The system L14H covers − 300 and + 200 along PC1 and − 200 and + 200 along PC2. The V28K PCA graph represents a region that lies between − 190 and + 200 along PC1 and between − 90 and + 190 along PC2.For the V28S system, the PCA plot covers − 450 and + 150 along PC1 and − 80 and + 200 along PC2.In contrast, regions − 250 and + 250 along PC1 and − 200 and + 180 along PC2 were covered by the wild-type system.

### Free energy landscape

**Figure 7:**
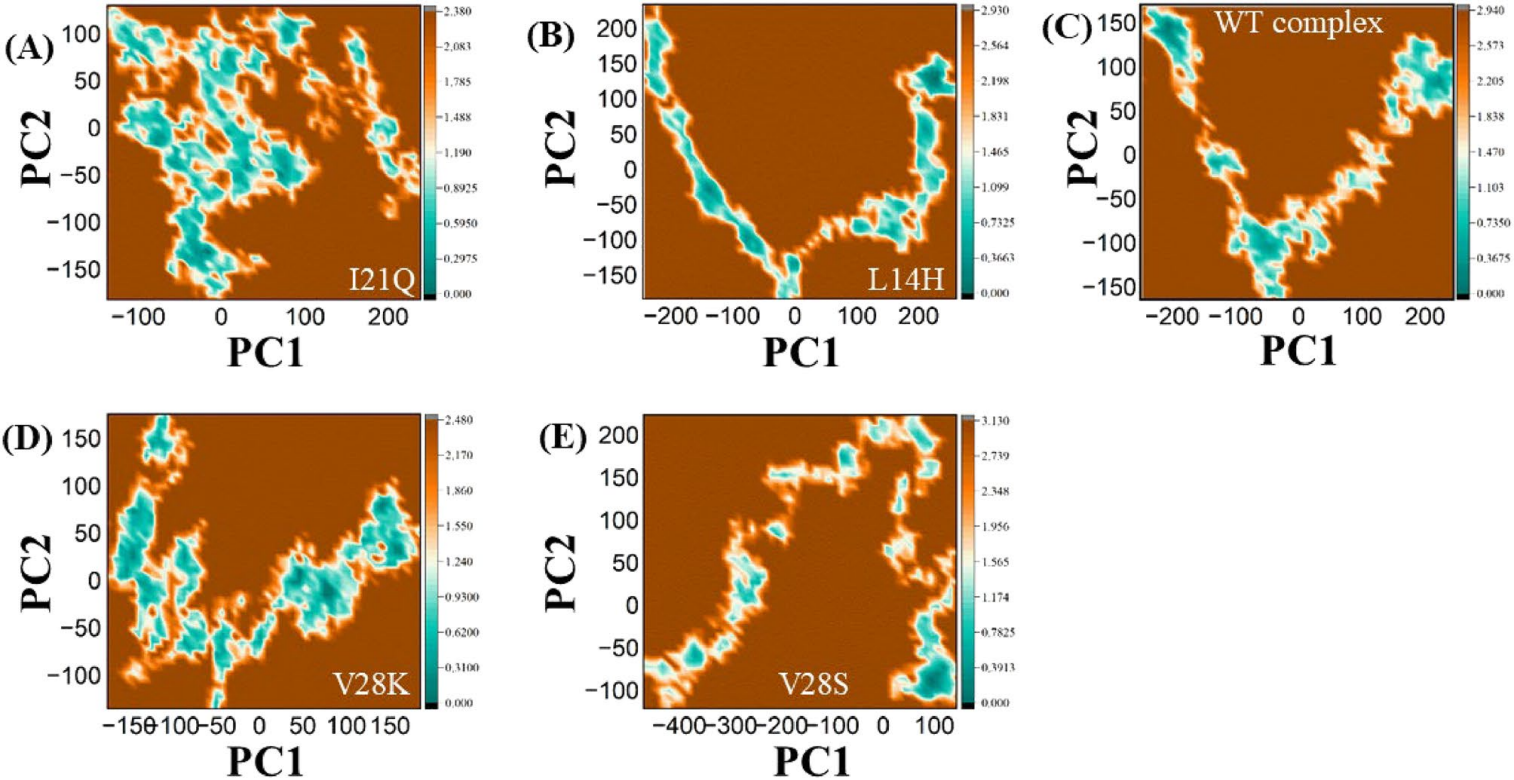
Free energy landscape (FEL) plots for the I21Q (**A**), L14H (**B**), and WT complex (**C**), and V28K (**D**) and V28S (**E**), peptide-protein complexes.

To further comprehend the variation of the stabilized conformation of the protein–ligand during the 100 ns simulation, the free energy morphology was mapped. Free energy topography was plotted using data from the three dimensions of RMSD, Rg (Radius of gyration), and Gibbs free energies, indicated by color codes from higher (red) to lower (blue) energies, and two types of free energy topography were plotted in 3D for a more intuitive presentation. In addition, the minimum energy conformations were extracted. Each complex has multiple conformations at the lowest energy, as demonstrated in the rightmost column of Fig. 7. In this analysis, the PCA (principal component analysis) is utilized to assess the free energy landscape, providing a more reliable presentation of the time and energy-dependent conformation space of proteins. Since, free energy landscape approach effectively differentiates between the kinetic and thermodynamic characteristics of proteins, rendering it a highly precise tool for comprehensive assessment of protein dynamics. The FEL plots for PC1 and PC2 exhibit variations in Gibbs free energy ranging from black to dark brown. This indicates a transition of protein energy from an unstable state with high energy to a more stable one with reduced energy. The conformational dynamics of PC1 and PC2 of the wild-type peptide and the top four design peptides are shown by FEL analysis. When compared to the wild-type reference peptide, the design peptide complexes, I21Q, L14H, V28K, and V28S, achieved low energy conformations and more stable behavior. Therefore, compared to the wild-type peptide, the binding of these design peptides is more favorable due to changes in conformation and dynamics, as shown by the results.

### MMGBSA analysis

The MMGBSA analysis was carried out for all the protein-peptide complexes and the result was compared with the wild-type complex. The binding energy calculation revealed that among all the systems the V28S peptide revealed strong binding toward the receptor. The delta G value of the V28S was good (− 51.7962) as compared to all other systems. The delta G value for the mutant system V28K was found as (− 50.9924) followed by L14H. As compared to the wild-type system the binding energy was good for the mutated systems. Table 3 represents the binding energy for all the systems.

**Table 3:** MMGBSA analysis of all the complexes.

S. no.	Peptide mutation	VDWAALS	EEL	ESURF	EGB	DELTA TOTAL kcal/mole
WT	0	− 61.1423	0.00	− 8.7600	187.1996	− 22.5538
1	I21Q	− 76.4065	− 147.6297	− 10.4922	209.0030	− 25.5499
3	L14H	− 97.2443	− 171.8324	− 13.1092	231.2084	− 50.9924
3	V28K	− 95.3670	− 154.8966	− 12.8590	213.9753	− 49.1672
4	V28S	− 100.1885	− 166.1172	− 13.7818	228.3103	− 51.7962

## Discussion

Millions of people have been infected by dengue fever all over the world. It is a disease that is transmitted by infected Aedes aegypti and Aedes albopictus mosquitoes. The infection can progress to the deadly dengue shock syndrome if ignored or not treated at the time^41^. Due to a lack of reliable vaccines and effective antiviral drugs, for dengue virus infections we aim to develop new peptide inhibitors for the dengue virus envelope protein. Because of their highly dynamic nature and enormous surface area, intracellular protein–protein interactions (PPIs) are challenging to target. PPI-targeted drug development techniques have made considerable progress and a number of drugs are presently available in the market while additional prospective PPI-targeting therapeutic compounds are being investigated in clinical trials^42,43^. Almost 645,000 PPI-related diseases are identified in humans^44^. Peptide drugs are less toxic and have better target selectivity in comparison to small compounds. By utilizing the information about protein–protein interactions, some scientists have created effective peptide inhibitors^45^. Over seven-thousand peptides with various bioactivities, such as anticancer, antifungal, antiviral, and antibacterial, were found in the previous ten years. The FDA has approved almost 60 peptide drugs and more than 500 that are now undergoing clinical trials^31^. The experimentally discovered peptide inhibitor DN59 was utilized in the current investigation to create novel peptide inhibitors using the computational framework. The native peptide inhibitor was docked with the target Envelope protein and then underwent an alanine scanning mutagenesis method to identify residues crucial for interactions. The findings were made about the stability and alteration of affinity when less significant residues involved in interactions were substituted with other residues. Mutations such as N8W, N8I, and N8Y leads to increase in the binding affinity of peptide toward the receptor and this result was confirmed by MD simulation. The MD simulation confirmed that N8W, N8I, N8Y, new peptide inhibitors revealed more stability and compactness towards Envelope receptor compared to the wild peptide inhibitor. In addition, more hydrogen bonds were observed in the new peptide inhibitors than the wild peptide during MD simulation. The results of the MMGBSA analysis, indicate that the new peptides proved to be potentially more effective than DN59. This is because these peptides have low binding energy value than the reference. Furthermore, it was found that the proposed mutant peptides can be used as peptide inhibitors that could lower the frequency of dengue infections.

## Conclusion

For designing peptide inhibitors against the dengue virus envelope protein a computational process including alanine scanning and residue scanning was carried out. A library of peptide inhibitors was developed using the residue scan analysis. The reference wild type and the top four peptides were subjected to MD simulation based on dAffinity scores. In addition, post-simulation studies were carried out, including PCA, RMSD, RMSF, Rg, and H-bond analysis. According to the binding free energies (MM/GBSA) of these developed peptides, the first peptide inhibitor was the most effective against the dengue virus envelope protein. All four of the suggested peptides had higher binding affinities for the envelope protein and have the potential to treat the dengue virus-associated infections.

### Supplementary Information

Supplementary Figures.

## Data Availability

Data will be available upon reasonable request from the corresponding author.
