# Supplementary material for: In silico design of peptide inhibitors for Dengue virus to treat Dengue virus-associated infections
Source: Sci Rep. 2024 Jun 7;14:13130. doi: 10.1038/s41598-024-63064-1 (PMC11161489; doi:10.1038/s41598-024-63064-1)
Supplement: Supplementary file 1 — Supplementary Figures. [file 41598_2024_63064_MOESM1_ESM.docx]

***Insilico* design of peptide inhibitors for Dengue virus to treat Dengue virus-associated infections**

Amar Ajmal^1#^, Muhammad Shahab^1,2#^, Muhammad Waqas^3^**,** Guojun Zheng^2^*, Maryam Zulfat^1^,Yousef A. Bin Jardan^4^, Gezahign Fentahun Wondmie^5^*, Mohammed Bourhia^6^, Ijaz Ali^7^

^1^ Department of Biochemistry, Abdul Wali Khan University Mardan.

^2^ State Key Laboratories of Chemical Resources Engineering, Beijing University of Chemical Technology, Beijing 100029, PR China.

^3^ Natural and Medical Sciences Research Center, University of Nizwa, Birkat Al-Mouz, Nizwa, 616, Oman

^4^ Department of Pharmaceutics, College of Pharmacy, King Saud University, P.O. Box 11451, Riyadh, Saudi Arabia

^5^ Department of Biology, Bahir Dar University, P.O.Box 79, Bahir Dar, Ethiopia

^6^ Department of Chemistry and Biochemistry, Faculty of Medicine and Pharmacy, Ibn Zohr University, Laayoune 70000, Morocco

^7^Centre for Applied Mathematics and Bioinformatics, Gulf University for Science and Technology, Hawally, Kuwait.

***Corresponding authors:** [zhenggj@mail.buct.edu.cn](mailto:zhenggj@mail.buct.edu.cn) (GZ); [resercherfent@gmail.com](mailto:resercherfent@gmail.com) (GFW)

^#=^ These authors contributed equally to this work

**Supplementary material**


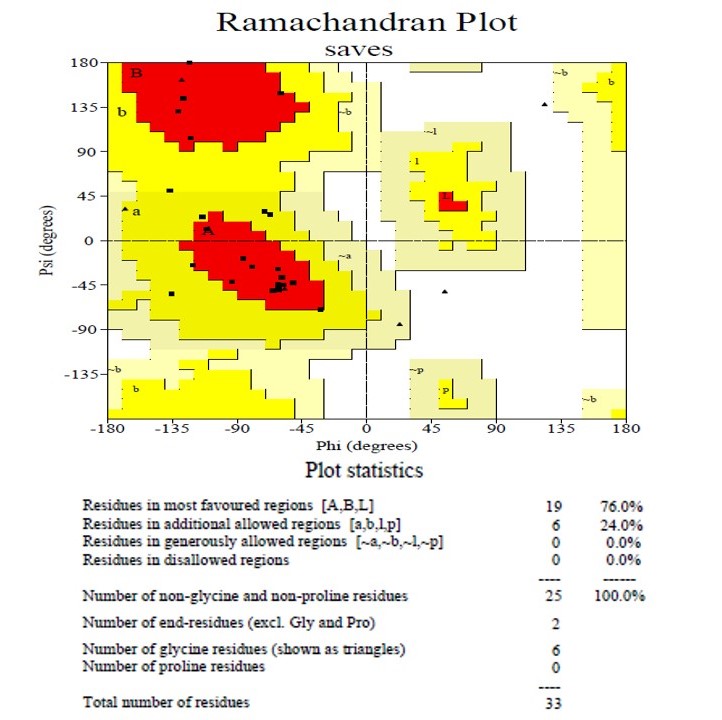


**Figure S1.** Ramachandran plot for the model developed by PepFold3 server.


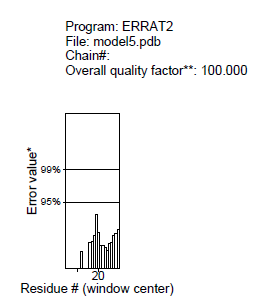


**Figure S2.** ERRAT plot for the developed model indicates an overall quality factor of 100.
